# Comprehensive Analysis of Immune Response and Transcriptome Profiling Reveals the Molecular Basis Underlying Breed-Specific Responses to *Eimeria tenella* Infection in Chickens with Differing Susceptibility Levels

**DOI:** 10.3390/ani15172533

**Published:** 2025-08-28

**Authors:** Jianmei Li, Haiyu Shen, Ming Xu, Wei Han, Xinhong Dou

**Affiliations:** 1 Key Laboratory for Poultry Genetics and Breeding of Jiangsu Province, Jiangsu Institute of Poultry Science, Yangzhou 225125, China; 2 Jiangsu Co-Innovation Center for Prevention and Control of Important Animal Infectious Diseases and Zoonoses, Yangzhou University, Yangzhou 225009, China

**Keywords:** Wenchang chicken, Recessive White Feather chicken, *Eimeria tenella*, immune response, RNA−seq

## Abstract

Wenchang Chickens (WCCs) and Recessive White Feather Chickens (RWFCs) are two important broiler breeds in China, although their susceptibility to *E. tenella* has not been compared. This study explores these differences in susceptibility. The results showed that WCCs exhibited lower susceptibility, as evidenced by no mortality and significantly reduced oocyst production compared to RWFCs. Additionally, WCCs had higher levels of CD3^+^CD4^+^ T lymphocytes and lower levels of CD3^+^CD8α^+^ T lymphocytes, both before and after infection, compared to RWFCs. Notably, serum IgA and IgG antibody levels in WCCs were significantly higher than those in RWFCs. RNA−seq analysis at 2, 4, and 7 days post-infection (dpi) revealed a consistent upward trend in gene expression in WCCs, while RWFCs exhibited a fluctuating pattern. Functional analysis indicated that the stable immune response, as annotated by the differentially expressed genes (DEGs) in WCCs, along with distinct metabolic alterations, may contribute to their enhanced resistance. Several hub genes, including SLC7A11, CCL19, CD4, HSPA5, and HSP90AA1, were identified within gene interaction networks specific to each breed. These findings provide valuable insights into the molecular mechanisms underlying the differential susceptibility of WCCs and RWFCs, offering potential targets for new coccidiosis control strategies.

## Simple Summary

It is well-established that different chicken breeds or lines exhibit varying susceptibilities to *Eimeria* infection. In this study, we compared the susceptibility of Wenchang Chickens (WCCs) and Recessive White Feather Chickens (RWFCs) to *Eimeria tenella* (*E. tenella*). We found that WCCs had lower susceptibility to infection, exhibiting stronger humoral and cellular responses than RWFCs. WCCs displayed consistently regulated genes associated with immune responses, while RWFCs exhibited upregulated gene expressions involved in lipid metabolism. Key genes like SLC7A11, CCL19, CD4, HSPA5, and HSP90AA1 may play a role in this difference. These findings offer new insights into coccidiosis prevention and improving chicken health and productivity.

## 1. Introduction

Chicken coccidiosis is an intestinal parasitic disease caused by *Eimeria* species, characterized by hemorrhagic diarrhea and dehydration [1]. Furthermore, *Eimeria* infection can make hosts more susceptible to opportunistic pathogens, such as *Clostridium*, *Campylobacter*, and *Salmonella*, significantly exacerbating clinical symptoms [2]. Among the seven *Eimeria* species commonly associated with avian coccidiosis, *Eimeria tenella*, which specifically parasitizes the cecum, is recognized as the most prevalent and pathogenic strain [3]. Currently, the reliance on vaccines and pharmaceutical interventions for the prevention and control of coccidiosis in poultry has notable limitations [4]. Therefore, it is crucial to develop novel strategies to prevent and control coccidiosis, addressing the current challenges effectively.

It has long been recognized that there are variations in susceptibility to *Eimeria* among different chicken breeds or lines. For instance, Fayoumi chickens are resistant to *E. tenella* infection, whereas Leghorn chickens are relatively more susceptible [5]. Specific haplotypes of the MHC-B and other alloantigen systems (D, E, and I) are associated with resistance and susceptibility traits during mixed *Eimeria* infection [6]. Du et al. [7] also found that different chicken breeds, such as yellow-feathered broilers, Arbor Acres broilers, and Lohmann pink layers, exhibit varying degrees of susceptibility to *E. tenella* infection. Additionally, Broadwater et al. [8] demonstrated that, in response to *E. maxima* infection, Fayoumi M5.1 chickens showed the least growth retardation, intestinal lesion scores, fecal oocyst shedding, and pathobiont proliferation compared to Ghs6 and Cobb chickens. This breed-specific response results from the co-evolutionary processes between the pathogen and host during prolonged interactions and is closely linked to the host’s genetic background, physiological and metabolic traits, and immune status [9,10]. Therefore, a comprehensive investigation into the mechanisms underlying variations in susceptibility to *Eimeria* across different host species is crucial for optimizing poultry management strategies, improving the efficacy of coccidiosis prevention and control measures, and advancing breed development.

Transcriptome sequencing technology, a cornerstone of modern biological research, allows for comprehensive and dynamic analysis of transcriptomic data within cells or tissues, offering valuable insights into the molecular mechanisms underlying disease susceptibility. Using RNA−seq, key differentially expressed mRNAs such as ANGPTL4, ACSL5, VEGFC, MAPK10, and CD44 have been identified in the cecal tissues of Jinghai yellow chickens following *E. tenella* infection [11]. RNA sequencing analysis also revealed distinct variations in the speed and intensity of cytokine transcriptional responses between the C.B12 and 15I lines of White Leghorn chickens, which exhibit differential susceptibility to *E. maxima* infection [4]. Furthermore, Kim et al. [12] utilized RNA sequencing to investigate the temporal dynamics of host responses in *Eimeria*-infected chickens, leading to the identification of key immunoregulatory hub genes and critical pattern recognition receptors. Collectively, these studies highlight the temporal and dynamic nature of host responses to *Eimeria*. However, the time-dependent gene expression profiles in different chicken breeds following *E. tenella* infection remain to be fully characterized.

Wenchang Chickens (WCCs), a distinctive native broiler breed in China, are known for their thin skin, tender bones, sweet and succulent meat, and exceptional resistance to stress and disease [13]. However, research on their susceptibility to specific pathogens remains limited. Recessive White Feather Chickens (RWFCs), originally from France, are fast-growing, white-feathered broilers. RWFCs have played a significant role in advancing high-quality chicken breeding programs and have greatly contributed to the development of China’s premium chicken industry. These two breeds represent important varieties in China’s broiler industry and are of considerable significance in the sector. Therefore, they may serve as appropriate experimental models for investigating potential differences in host susceptibility following *E. tenella* infection. In this study, we systematically investigated the differences in susceptibility between WCCs and RWFCs after *E. tenella* infection. The findings will enhance our understanding of host–pathogen interactions and provide valuable insights into the molecular basis of the differential susceptibility to *E. tenella* between WCCs and RWFCs.

## 2. Materials and Methods

### 2.1. Animals and Parasites

Seventy-one-day-old WCCs and seventy-one-day-old RWFCs, supplied by the National Gene Bank of Local Chicken Breeds (Jiangsu), were transported to the laboratory immediately after hatching and raised under standardized conditions in a coccidia-free environment. All experimental chickens remained unvaccinated and were provided with a complete, pathogen-free diet. Prior to the experiment, fecal samples from all chickens were subjected to microscopic examination using the saturated saline flotation technique to ensure the absence of *Eimeria* oocysts. Only chickens confirmed to be free from contaminating oocysts were used for the experiment. Throughout the entire experimental period, the chickens had ad libitum access to both feed and water.

The *E. tenella* Yangzhou strain (YZ) was isolated and maintained by the Parasitology Laboratory of the College of Veterinary Medicine, Yangzhou University. Prior to the experiment, the strain was reactivated in chickens, and the sporulated oocysts were subsequently collected [14]. The oocysts were then stored in a 2.5% potassium dichromate solution at 4 °C for subsequent use.

### 2.2. Experimental Design and Sample Collection

**Figure 1:**
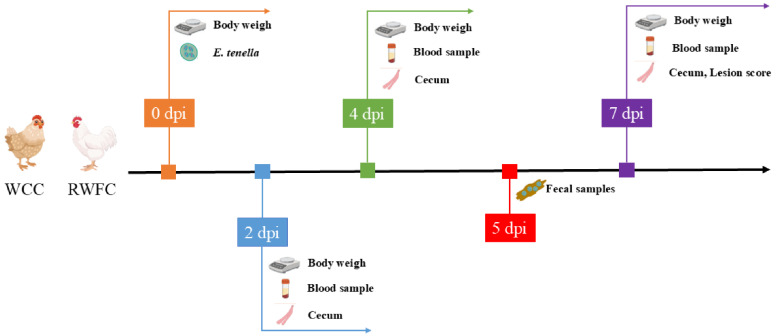
The experimental procedure and regimen of chickens.

The experimental procedure is shown in Figure 1. At 26 days of age, all experimental chickens were individually weighed and uniquely numbered. Sixty chickens of each breed with similar body conditions were selected and randomly divided into an infection group (3 replicates, 10 birds per replicate) and a non-infection control group (3 replicates, 10 birds per replicate). For the infection group, birds were orally administered 1 mL of sporulated oocyst suspension of *E. tenella* (containing 5 × 10^4^ oocysts), while the non-infection control group was inoculated with 1 mL of phosphate-buffered saline (PBS). After inoculation, daily observations were made regarding the chickens’ mental status, feed and water consumption, fecal characteristics, and any signs of disease. At 2, 4, and 7 days post-infection (dpi), three chickens were randomly selected from each group (one bird per replicate) for sampling. A total of 3 mL of blood was collected from each chicken using the aseptic cardiac puncture method. Of this, 2 mL was anticoagulated with 2% Na_2_EDTA for flow cytometry analysis. The remaining 1 mL was allowed to clot at room temperature, then centrifuged at 3000 rpm for 10 min to collect the serum, which was subsequently stored at −20 °C for future use.

Following blood sampling, chickens were euthanized using CO_2_ inhalation followed by cervical dislocation. Approximately 2 cm of cecal tissue was excised, and all tissue samples were coded. The tissues were then fixed in 4% paraformaldehyde solution for 24 to 48 h for subsequent H&E staining. The remaining cecal tissues were longitudinally sectioned, rinsed with sterile PBS, and stored at −80 °C for RNA extraction. Additionally, body weight was recorded one day prior to infection and at each sampling time point before culling. The McMaster counting method was used to quantify oocyst shedding in fecal samples collected from the infection groups between 5 to 7 dpi [15]. On day 7 post-infection, all remaining experimental chickens were sacrificed by CO_2_ inhalation and cervical dislocation. Cecal lesions were then evaluated and scored according to the methodology described by Johnson and Reid [16]. Differences in susceptibility to *E. tenella* between the two chicken breeds were assessed using mortality rate (MR), body weight gain (BWG), oocyst output (OPG) [15], and cecal lesion score (CLS) [16].

### 2.3. Peripheral Blood Lymphocyte Isolation and Flow Cytometric Analysis

Lymphocyte isolation was performed following the manufacturer’s protocol (P8740, Solarbio, Beijing, China), and then the cell density was adjusted to 1 × 10^6^ to 1 × 10^7^ cells/mL. Next, 300 μL of the diluted lymphocyte suspension was carefully aspirated and gently mixed with 2 μL of Mouse Anti-Chicken CD3-SPRD. This mixture was followed by the addition of 2 μL of either Mouse Anti-Chicken CD4-PACBLU or Mouse Anti-Chicken CD8α-AF700 (Southern Biotechnology Associates, Birmingham, AL, USA). The resulting mixture was incubated in the dark at 4 °C for 30 min, followed by washing with PBS to remove unbound antibodies. The labeled lymphocytes were then re-suspended in 300 μL of PBS and analyzed using a FACS Aria flow cytometer (BD Biosciences, San Jose, CA, USA). During the experiment, unmarked lymphocytes, as well as lymphocytes singly stained with CD3, CD4, or CD8, were used as controls. The procedural steps for the controls were synchronized with those of the experimental samples. Cells were analyzed using CytoFLEX 2.3.1.22 software (Beckman Coulter, Indianapolis, IN, USA), with lymphocytes identified based on forward scatter (FSC) and side scatter (SSC) gating. A total of 20,000 events within the specified gate were acquired for each sample. The results were subsequently analyzed to determine the percentage of CD3^+^CD4^+^ T lymphocytes and CD3^+^CD8α^+^ T lymphocytes.

### 2.4. ELISA Analysis of the Serum Antibody

The levels of IgG and IgA antibodies in serum were quantified using indirect ELISA. The optimal antigen coating concentration and serum dilution were determined by the checkerboard titration method. Each well was coated with 12.94 μg/mL of soluble sporozoite protein antigen and incubated at 37 °C for 2 h. The wells were then blocked with 5% skim milk at 4 °C overnight. The prepared chicken serum (diluted 1:160) and rabbit anti-chicken IgG-HRP (diluted 1:8000) (SE235, Solarbio, Beijing, China) were used as the primary and secondary antibodies, respectively. Both antibodies were incubated at 37 °C for 1 h. Tetramethylbenzidine (TMB) was added and incubated at 37 °C for 10 min, after which the reaction was terminated with 2 M H_2_SO_4_. The absorbance at 450 nm (OD_450_) was measured using a BioTek microplate reader (Epoch, BioTek, Winooski, VT, USA). The procedure for detecting IgA was identical to that for IgG, except that the secondary antibody was sheep anti-chicken IgA H&L (HRP) (ab112814, Abcam, Cambridge, UK). Serum from uninfected chickens was used as the negative control, while serum from chickens intensively immunized against *E. tenella* served as the positive control. In the ELISA, 100 μL of each sample was added to each well.

### 2.5. Total RNA Isolation, cDNA Library Construction, and Illumina Sequencing

RNA was extracted from the cecal tissues of WCCs and RWFCs infected with *E. tenella* at 2, 4, and 7 dpi using the RNeasy Mini Kit (Qiagen, Hilden, Germany). For each time point, three samples were collected from both the infection group and the non-infection control group, resulting in a total of 36 samples. RNA concentration and purity were assessed using the NanoDrop One micro-volume UV-Vis spectrophotometer (Thermo Scientific, Waltham, MA, USA), while RNA integrity was evaluated using the Agilent 4200 TapeStation (Agilent, Santa Clara, CA, USA). Only samples with an OD260/280 ratio greater than 1.8 and an RNA integrity number (RIN) greater than 7 were selected for library construction and deep sequencing (Table A1).

After purification of total RNA (>2 μg per sample), mRNA was enriched using magnetic beads conjugated with Oligo (dT) (NEBNext^®^ Poly(A) mRNA Magnetic Isolation Module, New England Biolabs, Ipswich, MA, USA). The enriched mRNA was then fragmented using the fragmentation buffer provided in the NEBNext^®^ Ultra^TM^ RNA Library Prep Kit for Illumina^®^ (New England Biolabs, Ipswich, MA, USA) to generate short fragments. First-strand cDNA synthesis was performed using random hexamer primers, followed by second-strand synthesis using DNA polymerase I, RNase H, and dNTPs supplied in the same kit. The double-stranded cDNA was purified using AMPure XP beads (Beckman Coulter, Brea, CA, USA), end-repaired, A-tailed, and ligated with Illumina sequencing adapters (NEBNext^®^ Multiplex Oligos for Illumina, New England Biolabs, Ipswich, MA, USA). Small fragment sequencing libraries were constructed for each sample according to the manufacturer’s protocol. Paired-end (PE150) sequencing was then performed on the Illumina NovaSeq 6000 platform (Illumina, San Diego, CA, USA).

The raw sequencing data underwent quality control analysis using FastQC software (version 0.10.1), as detailed by Chen et al. [17]. Trimmomatic (v0.35) software was used to remove reads containing adapters, sequences with ambiguous bases denoted by ‘N’, low-quality reads where more than 50% of the bases had a quality score ≤ 20, and reads shorter than 30 nucleotides. Following this, rRNA reads were filtered out using Bowtie2 to obtain clean reads. The clean reads were then aligned to the chicken reference genome (Gallus gallus, GRCg6a, GCA_000002315.5) using Hisat2 [18]. The reference genome and annotation files were retrieved from the Ensembl Genome Browser FTP site (ftp://ftp.ensembl.org/pub/release-104/fasta/gallus_gallus/dna/, accessed on 6 March 2023). The aligned reads were assembled and quantified using StringTie [19]. Subsequently, the Trimmed Mean of M-values (TMM) normalization algorithm was applied to normalize the read counts, and FPKM values were calculated. The data have been deposited in the NCBI Sequence Read Archive (SRA) database. The BioProject accession numbers are as follows: for RWFC breed samples, PRJNA1203686 (2 dpi), PRJNA1205407 (4 dpi), and PRJNA1205890 (7 dpi); for WCC breed samples, PRJNA1209259 (2 dpi), PRJNA1210681 (4 dpi), and PRJNA1212294 (7 dpi).

### 2.6. Differentially Expressed Genes (DEGs) and Functional Enrichment Analysis

DEGs between the infected and non-infected control groups of WCC and RWFC breeds were analyzed at three post-infection time points (2, 4, and 7 dpi) using the R software package edgeR (version 3.30.0). The analysis was performed based on FPKM values, *p*-values, and fold change (FC). Specifically, comparisons were made between WCC−T vs. WCC−C and RWFC−T vs. RWFC−C, as well as between WCC−Tand RWFC−T, to identify unique DEGs. A significance threshold was set at a fold change ≥ 1.5 and an adjusted *p*-value < 0.05. Following this, ClusterProfiler [20] was used to perform Gene Ontology (GO) functional annotation and Kyoto Encyclopedia of Genes and Genomes (KEGG) pathway enrichment analysis on the specific DEGs identified between the two breeds. GO terms and KEGG pathways with an adjusted *p*-value < 0.05 were considered significantly enriched.

### 2.7. Construction of Gene–Gene Interaction Networks

Based on the Pearson correlation coefficient, gene–gene interaction networks of the unique DEGs between WCCs and RWFCs were constructed at three time points following infection. The network construction procedure is as follows. (1) Data preprocessing. For different transcripts of the same gene, the median expression value was used as the representative gene expression value, with no additional processing applied. (2) Data screening. Differentially expressed mRNA profiles were extracted from the WCC and RWFC datasets. (3) Calculation of Pearson correlation coefficients. R packages were used to compute the correlation coefficients for the specific DEGs in WCCs and RWFCs separately. (4) Correlation coefficient filtering. A significance threshold was set at a Pearson’s correlation coefficient ≥ 0.80 or ≤−0.80, with *p*-values < 0.05. The gene–gene interaction networks were then constructed and visualized using OmicShare tools (http://www.omicshare.com/tools/Home/Soft/cytoscape, accessed on 8 July 2025) [21]. Finally, hub genes with high connectivity in the network were identified for further analysis.

### 2.8. Analysis of the Overlapping Genes at Three Time Points

The overlapping DEGs between the infection groups and non-infection control groups of WCCs and RWFCs at three post-infection time points (2, 4, and 7 dpi) were analyzed separately. Subsequently, on the Omicsmart platform (https://www.omicsmart.com, accessed on 10 January 2025), the Mfuzz package was used for fuzzy c-means clustering to classify the intersecting genes from both chicken breeds into distinct clusters for trend analysis. [22]. Additionally, GO and KEGG pathway enrichment analyses were performed on the overlapping DEGs, with *p*-values < 0.05 indicating statistically significant enrichment of associated terms and pathways. Finally, based on the Pearson correlation coefficient, gene–gene interaction networks were constructed for the overlapping genes at the three post-infection time points in both WCCs and RWFCs. Hub genes were then identified from these networks to uncover key regulatory elements.

### 2.9. Validation of the RNA−seq Data via qRT−PCR

To assess the reliability of the transcriptome sequencing results, 20 unique DEGs were selected from WCCs and RWFCs for further validation. This selection included 10 genes from WCCs and 10 from RWFCs, which were subsequently subjected to quantitative reverse transcription polymerase chain reaction (qRT−PCR) verification. Gene-specific primers for qRT−PCR analysis were designed using the NCBI Primer-BLAST tool (https://www.ncbi.nlm.nih.gov/tools/primer-blast/, accessed on 12 June 2023). (Table A2). Total RNA was extracted from cecal samples using the TaKaRa MiniBEST Universal RNA Extraction Kit (9767, TaKaRa, Dalian, China), and cDNA was synthesized using the PrimeScript RT Reagent Kit with gDNA Eraser (Perfect Real Time) (RR047A, TaKaRa, Dalian, China). qRT−PCR was performed using TB Green Premix Ex Taq II (Tli RNaseH Plus) (RR820Q, TaKaRa, Dalian, China) on the Mx3000P real-time PCR system (Agilent Technologies, Palo Alto, CA, USA). The reaction program included 95 °C for 30 s, followed by 40 cycles of 95 °C for 10 s and 60 °C for 30 s. A melting curve analysis was performed at 95 °C for 15 s, 60 °C for 60 s, and 95 °C for 15 s. GAPDH was used as the internal control gene, with each sample tested in triplicate. The relative expression levels of genes were calculated using the 2^−ΔΔCt^ method [23]. Finally, a log2 transformation was applied to the expression values of each gene obtained from both qRT−PCR and RNA−seq analysis.

### 2.10. Data Statistical Analysis

The experimental data were analyzed using SPSS Statistics 22.0 software (IBM Inc., Armonk, NY, USA), and the results were presented as mean ± standard error (SE). For pairwise comparisons between the infection and control groups of the same breed at the same time point, as well as between different breeds, Student’s *t*-test was used for significance analysis. For comparisons within the same breed at the three post-infection time points, one-way ANOVA was performed to compare group means, followed by Duncan’s multiple range test for further inter-group analysis. Additionally, given the ordinal nature of the cecal lesion score data, non-parametric testing methods (Kruskal–Wallis test) were employed for statistical analysis. A *p*-value < 0.05 was considered statistically significant for all analyses. Bar charts were generated using GraphPad Prism version 9.0 (GraphPad Software, San Diego, CA, USA). Volcano plots, heatmaps, GO enrichment maps, and KEGG pathway maps were created using the online platform https://www.bioinformatics.com.cn (accessed on 17 January 2025), a specialized tool for data analysis and visualization [24].

## 3. Results

### 3.1. Different Susceptibility to E. tenella Infection Between WCCs and RWFCs

**Figure 2:**
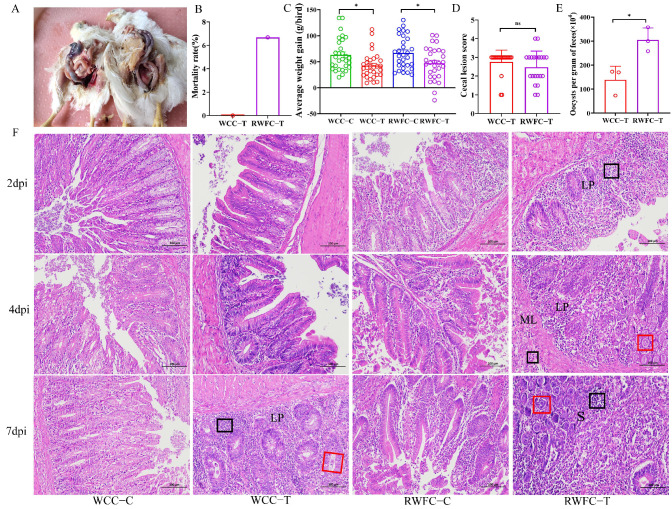
Clinical symptoms of WCCs and RWFCs infected with *E. tenella*. (**A**) Two RWFCs died at 5 dpi. (**B**) Mortality rate statistics were recorded for both chicken breeds. (**C**) Average weight gain of the two chicken breeds. (**D**) Cecal lesion scores of the two chicken breeds. (**E**) Oocyst count per gram of feces from the two chicken breeds. (* *p* < 0.05; *ns*, not significant). (**F**) Panels representing pathological changes in each group at 2, 4, and 7 dpi. No significant inflammatory cell infiltration was observed in the ceca of uninfected WCCs (WCC−C) and uninfected RWFCs (RWFC−C). However, lymphocyte infiltration (shown by black boxes) was evident in the ceca of infected WCCs (WCC−T) and infected RWFCs (RWFC−T), along with schizonts or oocysts (shown by red boxes). Abbreviations: LP, lamina propria; ML, muscularis mucosae; S, submucosa. (*n* = 3, H&E staining, magnification ×200.)

To assess the differences in susceptibility to *E. tenella* between WCCs and RWFCs, we systematically evaluated the MR (mortality rate), BWG (body weight gain), OPG (oocyst production), and CLS (cecal lesion score) following infection. Throughout the entire experimental period, both the non-infected group and the WCC-infected (WCC−T) group maintained a 100% survival rate. In contrast, mortality in the RWFC-infected (RWFC−T) group began on the 5th day post-infection (dpi), with an MR of 6.67% (two out of thirty chickens) (Figure 2A,B). Regarding BWG, infected birds in both groups showed significantly lower weight gain compared to the uninfected controls (Figure 2C). Notably, two birds from the RWFC−T group experienced significant weight loss and died by 5 dpi. The CLS analysis revealed no significant differences between the WCC−Tand RWFC−T groups (Figure 2D). However, during the peak oocyst production period, the WCC−T group exhibited significantly lower oocyst yield than the RWFC−T group (Figure 2E).

Pathological analysis of cecal tissues revealed no significant infiltration of inflammatory cells in the WCC−T group at 2 or 4 dpi. By 7 dpi, occasional focal lymphocytic infiltration (black boxes) and a few oocysts (red boxes) were observed in the lamina propria. In contrast, the RWFC−T group showed an inflammatory response as early as 2 dpi, with moderate lymphocytic infiltration (black boxes). By 4 dpi, inflammation in RWFC−T birds intensified, with inflammatory cells infiltrating the muscular layer (black boxes) and a substantial presence of schizonts and oocysts (red boxes) (Figure 2F). Altogether, these findings suggest that WCC exhibit lower susceptibility to *E. tenella* compared to RWFCs.

### 3.2. E. tenella-Induced Alterations of CD3^+^CD4^+^ and CD3^+^CD8α^+^ T lymphocyte Subsets in WCCs and RWFCs

**Figure 3:**
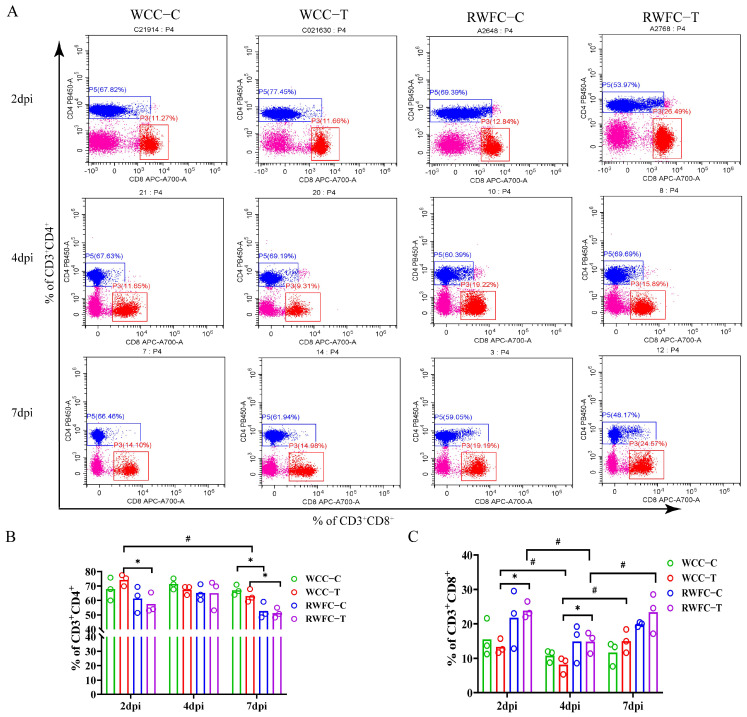
Changes in CD3^+^CD4^+^ and CD3^+^CD8α^+^ T cell populations following WCC and RWFC infection with *E. tenella*. The percentages of CD3^+^CD4^+^ and CD3^+^CD8α^+^ T cells were assessed at 2, 4, and 7 dpi using FACS. (**A**) Panels illustrating the distributions of CD3^+^CD4^+^ and CD3^+^CD8α^+^ T cells for each group at the specified time points (2, 4, and 7 dpi). Cells shown in pink represent CD3⁺ T lymphocytes that are negative for both CD4 and CD8α. (**B**) Percentages of CD3^+^CD4^+^ T cells within peripheral blood T lymphocytes were quantified via FACS analysis. (**C**) Percentages of CD3^+^CD8α^+^ T cells in peripheral blood T lymphocytes were determined using FACS. Unpaired *t*-tests were conducted to evaluate the differences between the control and infection groups within the same breed at identical time points, as well as the inter-group differences between the WCC−T and RWFC−T groups and between the WCC−C and RWFC−C groups at corresponding time points (* *p* < 0.05). One-way ANOVA followed by Dunnett’s multiple comparisons test was utilized to evaluate differences between infected groups versus their respective controls at 2, 4, and 7 dpi (^#^ *p* < 0.05).

To investigate the differences in cellular immune responses between WCCs and RWFCs following infection with *E. tenella*, changes in CD3^+^CD4^+^ and CD3^+^CD8α^+^ T lymphocyte subsets were analyzed by flow cytometry (FACS). The results demonstrated that, at 2 dpi, the proportion of CD3^+^CD4^+^ T lymphocytes was significantly higher in the WCC−T group compared to the RWFC−T group (Figure 3A,B), whereas the proportion of CD3^+^CD8α^+^ T lymphocytes was significantly lower in the WCC−T group than in the RWFC−T group. (Figure 3A,C). At 4 dpi, the WCC−T group still exhibited a significantly lower count of CD3^+^CD8α^+^ T lymphocytes than the RWFC−T group. At 7 dpi, the WCC−T group exhibited a significantly higher proportion of CD3^+^CD4^+^ T lymphocytes compared with the RWFC−T group. Furthermore, when comparing different time points, at 2 dpi, the WCC−T group had a significantly higher level of CD3^+^CD4^+^ T lymphocytes than at 7 dpi. Additionally, at both 2 and 7 dpi, the levels of CD3^+^CD8α^+^ T lymphocytes in both the WCC−T and RWFC−T groups were markedly elevated compared to those at 4 dpi (Figure 3B,C). Taken together, these results highlight distinct patterns of T cell−mediated immune responses between WCCs and RWFCs during *E. tenella* infection.

### 3.3. E. tenella-Induced Alterations of Serum IgG and IgA in WCCs and RWFCs

**Figure 4:**
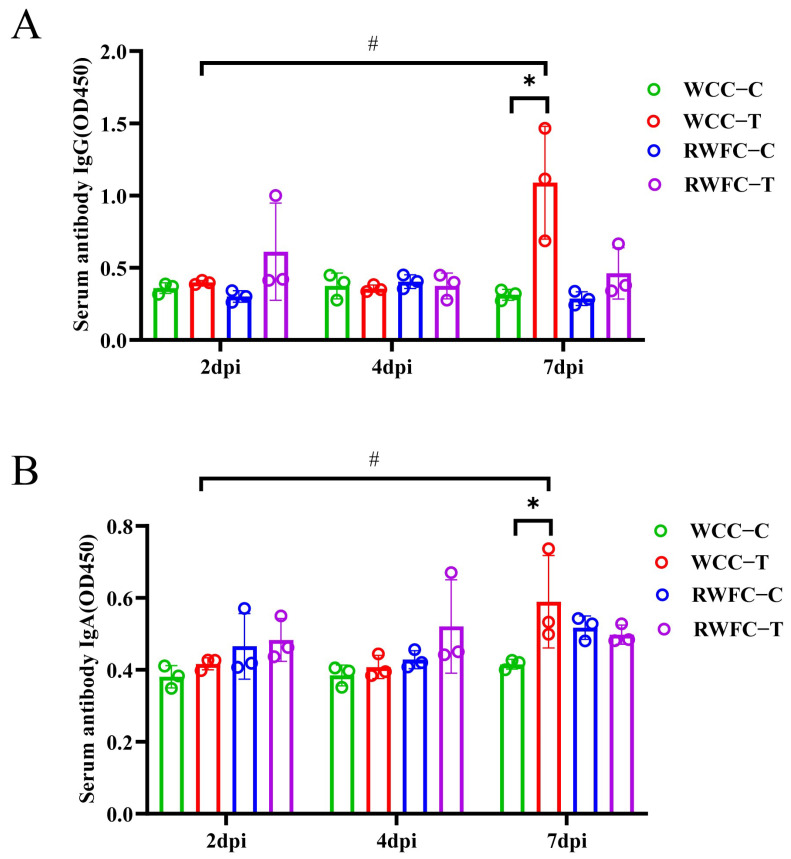
Levels of IgG and IgA antibodies in WCCs and RWFCs following *E. tenella* infection. (**A**) Measurement of serum IgG levels in WCCs and RWFCs at 2, 4, and 7 dpi using ELISA. (**B**) Measurement of serum IgA levels in WCCs and RWFCs at 2, 4, and 7 dpi utilizing ELISA techniques. An unpaired *t*-test was utilized to evaluate the differences between the control groups and their corresponding infected groups of the same breed at each time point, as well as to compare the differences between WCC−Tand RWFC−T, and between WCC−C and RWFC−C at identical time points (* *p* < 0.05). A one-way ANOVA followed by Dunnett’s multiple comparisons test was utilized to evaluate the differences between the infected groups and their respective control groups at 2, 4, and 7 dpi (^#^ *p* < 0.05).

To assess the differences in humoral immune responses between WCCs and RWFCs following *E. tenella* infection, serum IgG and IgA antibody levels were measured using an indirect ELISA assay. At 2 dpi, both the WCC−T and RWFC−T groups showed an upward trend in IgG levels compared to their respective control groups (Figure 4A), although the differences were not statistically significant. By 7 dpi, IgG levels in both infected groups had increased relative to their respective controls; however, a statistically significant elevation was observed only in the WCC−T group. Moreover, IgG levels in the WCC−T group at 7 dpi were significantly higher than those recorded at 2 and 4 dpi. The pattern of IgA antibody changes mirrored that of IgG (Figure 4B). More specifically, at 2 and 4 dpi, IgA levels in both the WCC−Tand the RWFC−T groups were higher than those in their respective control groups. At 7 dpi, IgA levels in the WCC−T group were significantly greater than those in the uninfected control group and markedly higher than the levels at 2 and 4 dpi. In contrast, IgA levels in the RWFC−T group remained relatively stable throughout the infection period. Overall, these results indicate that WCC−T chickens mount a stronger humoral immune response than RWFC−T chickens during *E. tenella* infection.

### 3.4. RNA−seq Profiles of WCC and RWFC Cecum Tissues Following E. tenella Infection

To investigate the potential mechanisms underlying the differences in immune responses to *E. tenella* infection between WCCs and RWFCs, RNA−seq analysis was performed on cecal tissues from both breeds at 2, 4, and 7 dpi. In total, 36 RNA−seq libraries were constructed and sequenced (Table A3). The total number of raw reads generated was 826,318,548 for WCCs and 804,497,866 for RWFCs. After removing adapter sequences and low-quality reads, each library yielded an average of 44,765,911 clean reads, with Q30 values exceeding 92.94%. These clean reads were then aligned to the chicken reference genome (GRCg6a), achieving an average alignment rate of 92.17% (Table A4). Collectively, these results confirm that all libraries produced high-quality clean reads suitable for downstream analyses.

### 3.5. Analysis of Gene Expression in Cecal Tissues of WCCs and RWFCs Following E. tenella Infection

**Figure 5:**
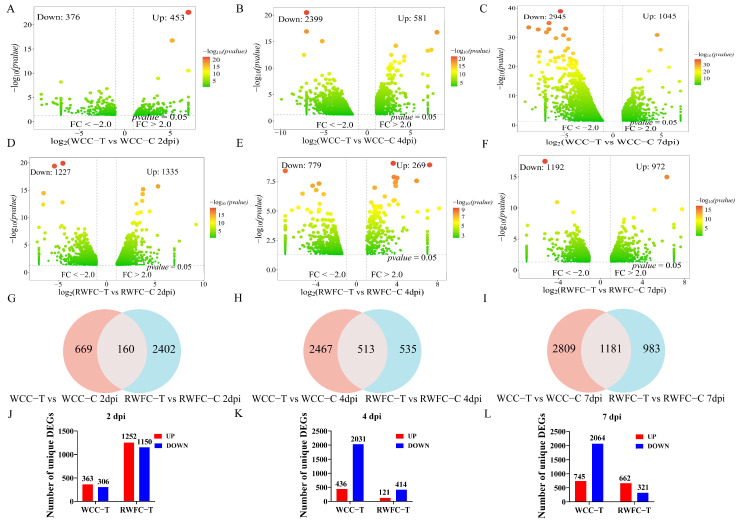
The number of DEGs in WCCs and RWFCs at 2, 4, and 7 dpi. (**A**–**C**) Volcano plots illustrating the comparison of DEGs between WCC−Tand WCC−Cat 2 dpi (**A**), 4 dpi (**B**), and 7 dpi (**C**). (**D**–**F**) Volcano plots depicting the comparison of DEGs between RWFC−Tand RWFC−Cat 2 dpi (**D**), 4 dpi (**E**), and 7 dpi (**F**). The numbers of significantly downregulated and upregulated DEGs were shown at the top of each plot. (**G**–**I**) Venn diagram analyses comparing WCC−T between and RWFC−Tat 2 dpi (**G**), 4 dpi (**H**), and 7 dpi (**I**). (**J**–**L**) Number of specifically expressed DEGs in WCC−T relative to RWFC−Tat 2 dpi (**J**), 4 dpi (**K**), and 7 dpi (**L**).

RNA−seq analysis revealed distinct temporal patterns in differential gene expression between the two breeds. At 2 dpi, the number of DEGs in RWFCs (2562) was markedly higher than that in WCCs (829) when compared with their respective control groups (Figure 5A,D). In contrast, at 4 and 7 dpi, WCCs exhibited more DEGs than RWFCs. Notably, within WCCs at both 4 and 7 dpi, downregulated DEGs consistently outnumbered upregulated ones (Figure 5B,C,E,F). Analysis of overlapping DEGs between the two breeds showed a gradual increase across the three time points (Figure 5G–I), suggesting that these shared genes may play a role in the common host response to *E. tenella* infection. We then compared the unique DEGs between the WCC−Tand RWFC−T groups (Figure 5J–L). At 2 dpi, the WCC−T group had 669 unique DEGs (363 upregulated, 306 downregulated), while the RWFC−T group displayed 2402 unique DEGs (1252 upregulated, 1150 downregulated) (Figure 5J). At 4 dpi, the number of unique DEGs in the WCC−T group rose sharply to 2467 (436 upregulated, 2031 downregulated), whereas the RWFC−T group declined to 535 unique DEGs (121 upregulated, 414 downregulated) (Figure 5K). At 7 dpi, the WCC−T group continued to show more unique DEGs than the RWFC−T group (Figure 5L). Overall, these results indicate that following *E. tenella* infection, WCCs display a steadily increasing DEG profile, whereas RWFCs exhibit a more variable, fluctuating pattern. The expression dynamics of the unique DEGs in each breed closely mirrored the overall DEG expression trends.

### 3.6. Functional Annotation of the Unique DEGs in WCCs and RWFCs Following E. tenella Infection

To investigate the functions of the unique DEGs induced in WCCs or RWFCs following *E. tenella* infection, we performed GO and KEGG enrichment analyses.

KEGG pathway analysis

**Figure 6:**
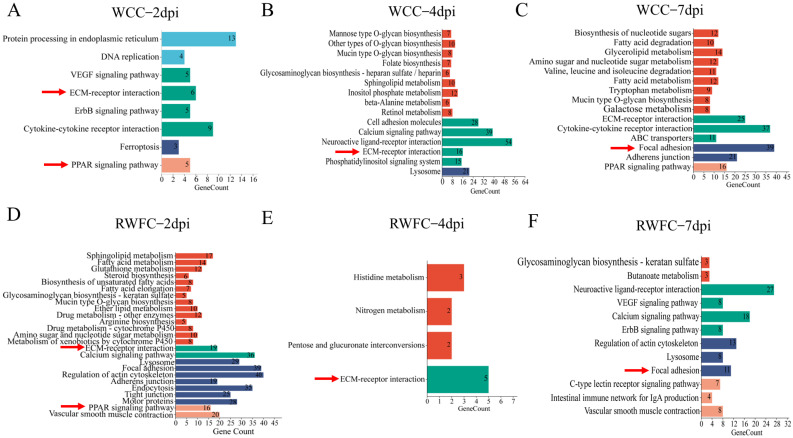
KEGG pathway enrichment analysis of WCC–specific and RWFC–specific DEGs following *E. tenella* infection. (**A**–**C**) Illustrates the significantly enriched pathways for WCC–specific DEGs at 2 dpi (**A**), 4 dpi (**B**), and 7 dpi (**C**). (**D**–**F**) Depicts the significantly enriched pathways for RWFC–specific DEGs at 2 dpi (**D**), 4 dpi (**E**), and 7 dpi (**F**). At each time point, pathways common to both breeds are marked with red arrows. In the pathway diagrams, red represents metabolism-related pathways, green represents environmental information processing-related pathways, blue represents cellular processes-related pathways, and orange represents organismal systems-related pathways. The specific DEGs analyzed in this study were derived from comparisons between the WCC−T group and the RWFC−T group.

At 2 dpi, WCC–specific DEGs were significantly enriched in eight pathways, five of which were associated with environmental information processing (Figure 6A). In contrast, RWFC–specific DEGs were enriched in 25 pathways, with 14 associated with metabolic processes (Figure 6D). At 4 dpi, the number of enriched pathways in WCCs increased to 15, primarily involving metabolic pathways (9) and environmental information processing (5) (Figure 6B), whereas RWFC enrichment dropped sharply to four pathways, three of which were metabolic (Figure 6E). By 7 dpi, WCCs maintained enrichment in fifteen pathways (nine metabolic), while RWFC enrichment rose to twelve pathways (Figure 6C,F).

KEGG dynamic analysis revealed that metabolic pathways induced by WCC–specific DEGs showed a progressive increase, accompanied by upward trends in environmental information processing and cellular processes. In contrast, metabolic pathways in RWFC–specific DEGs generally declined over time. Despite these differences, both breeds shared enrichment in several pathways, including ECM–receptor interaction, PPAR signaling, and focal adhesion. Overall, WCC–specific DEGs demonstrated a progressive increase in both the number of enriched KEGG pathways and associated genes, while RWFC–specific enrichment followed a rise–fall–rise pattern. Notably, several key pathways enriched in WCC–specific DEGs, including protein processing in the endoplasmic reticulum, cytokine–cytokine receptor interaction, ferroptosis, and multiple glycan, lipid, and amino acid metabolic pathways, may contribute to the higher resistance of WCCs to *E. tenella*.

GO term analysis

**Figure A1:**
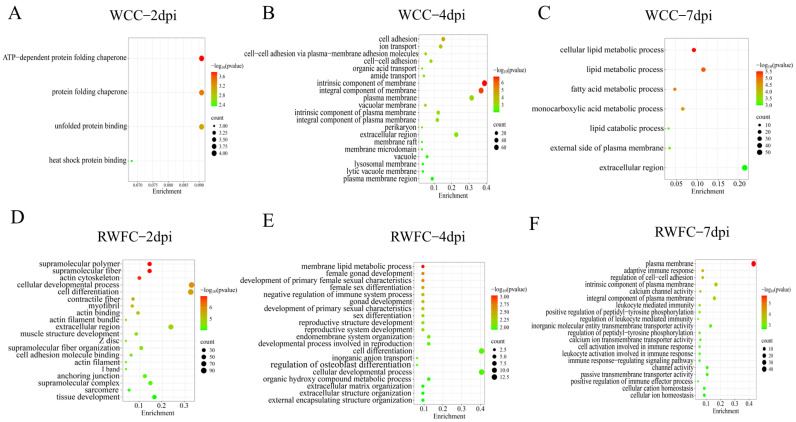
The GO enrichment analysis was conducted on the unique DEGs in the cecal tissues of WCCs and RWFCs during *E. tenella* infection. (**A**–**C**) Present the GO term analysis for the WCC–specific DEGs at 2 dpi (**A**), 4 dpi (**B**), and 7 dpi (**C**), respectively. (**D**–**F**) Provide the GO term analysis for the RWFC–specific DEGs at 2 dpi (**D**), 4 dpi (**E**), and 7 dpi (**F**), respectively. The unique DEGs analyzed herein represent a comparison between the WCC−T and RWFC−T groups.

At 2 dpi, only four GO terms—belonging to the molecular function (MF) category—were significantly enriched among WCC–specific DEGs (Figure A1A). In contrast, RWFC–specific DEGs were enriched in 84 GO terms, with the top 20 mainly in the cellular component (CC) category (Figure A1D). At 4 dpi, WCC–specific enrichment rose to 30 GO terms, primarily in biological processes (BPs) and CCs (Figure A1B), whereas RWFC–specific DEGs were enriched in 85 GO terms, with all top 20 belonging to BPs (Figure A1E). By 7 dpi, WCC–specific DEGs were enriched in only seven GO terms, mainly BPs related to lipid metabolism (Figure A1C), while RWFC–specific DEGs were enriched in 87 GO terms, again dominated by BPs (Figure A1F). Overall, GO analysis demonstrated clear differences between breeds. Specifically, WCC–specific GO terms followed a fluctuating pattern—initially increasing and then decreasing—whereas RWFC–specific enrichment remained relatively stable. Importantly, at 7 dpi, WCC–specific DEGs were primarily associated with lipid metabolism, which may be linked to their greater resistance to *E. tenella*.

### 3.7. Gene–Gene Interaction Network of WCC–Specific and RWFC–Specific DEGs Following E. tenella Infection

To further explore the underlying anti-infection mechanisms following *E. tenella* infection, we identified the unique DEGs based on GO terms and KEGG pathways for both WCC and RWFC breeds. Gene–gene interaction networks were then constructed for each breed at three distinct post-infection time points (2, 4, and 7 dpi). Additionally, unique DEGs were selected from these networks for cluster analysis.

**Figure 7:**
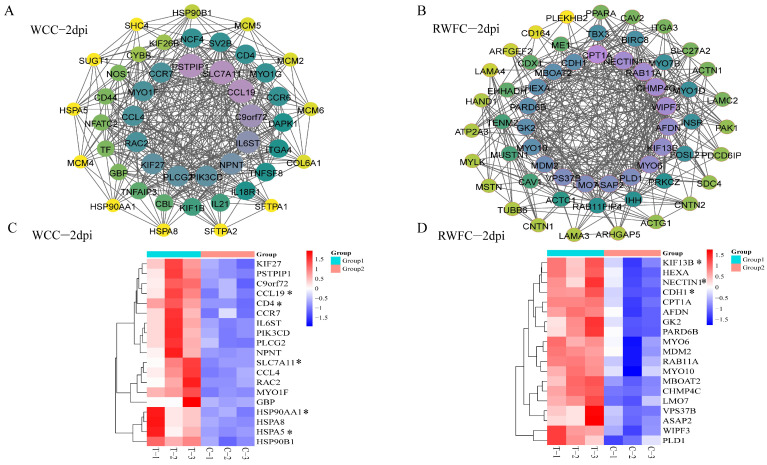
Gene–gene interaction network and heatmap analysis of the WCC–specific and RWFC–specific DEGs at 2 dpi. (**A**) Gene–gene interaction network of the selected WCC–specific DEGs. (**B**) Gene–gene interaction network of the selected RWFC–specific DEGs. (**C**) Heatmaps depicting selected genes from the gene–gene interaction network of WCC–specific DEGs. (**D**) Heatmaps depicting selected genes from the gene–gene interaction network of RWFC–specific DEGs. The dimensions and hues of the circles reflect the magnitude of the degree scores. Furthermore, the data visualization conventions in Figure A2, Figure A3 and Figure A4 adhered to the same principles. The genes highlighted with asterisks in the heatmap will be utilized for subsequent qRT−PCR validation experiments.

At 2 dpi, the gene–gene interaction network for WCC–specific DEGs comprised 46 nodes. Notably, the upregulated genes SLC7A11, CCL19, PSTPIP1, C9orf72, IL6ST, and NPNT exhibited connectivity greater than 20 nodes (Figure 7A). In contrast, the RWFC–specific network contained 59 nodes, with upregulated genes such as NECTIN1, WIPF3, and CPT1A targeting more than 20 nodes (Figure 7B). Cluster analysis of the unique DEGs from the gene–gene interaction networks revealed a clear differential expression pattern between the WCC–specific and RWFC–specific DEGs, as shown in Figure 7C,D, where the expression levels are represented by varying intensities of red and blue.

**Figure A2:**
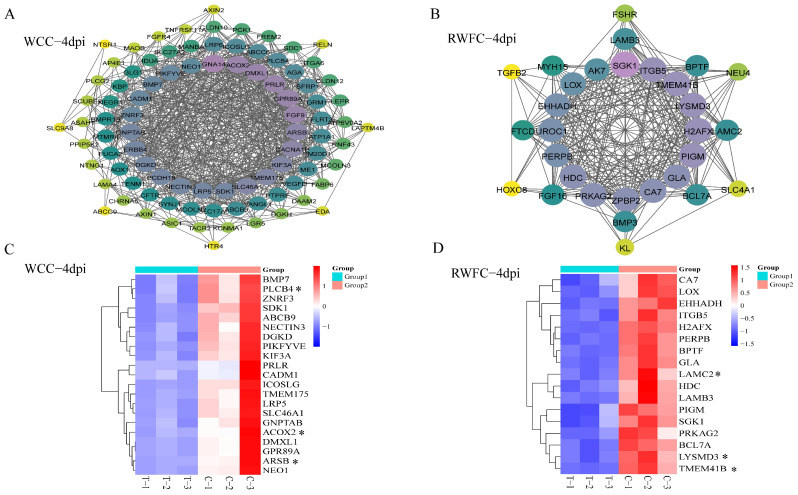
Gene–gene interaction network and heatmap analysis of the WCC–specific DEGs and RWFC–specific DEGs at 4 dpi. Genes marked with asterisks in the heatmap were selected for subsequent qRT−PCR validation.

**Figure A3:**
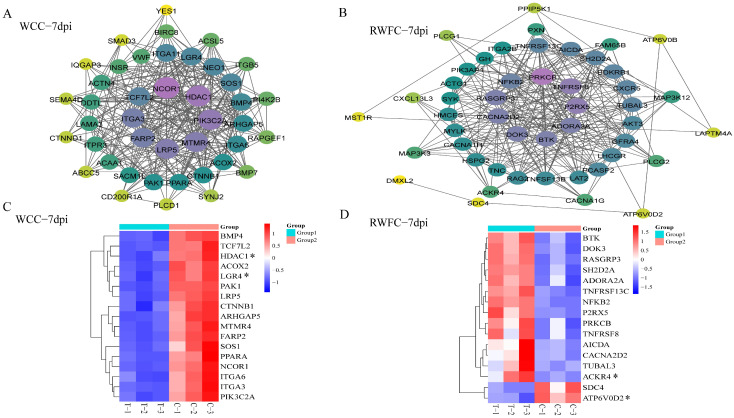
Gene–gene interaction network and heatmap analysis of the WCC–specific DEGs and RWFC–specific DEGs at 7 dpi. Genes marked with asterisks in the heatmap were selected for subsequent qRT−PCR validation.

At 4 dpi, the WCC–specific gene–gene interaction network grew to 90 nodes (Figure A2A), with genes such as GNA14, GPR89A, DMXL1, ACOX2, FGF9, and PRLR targeting more than 25 nodes. In contrast, the RWFC–specific network shrank to 30 nodes (Figure A2B), with only SGK1 targeting more than 25 nodes. The cluster heatmap showed that overall expression of the unique DEGs in both breeds was downregulated following *E. tenella* infection (Figure A2C,D). At 7 dpi, the WCC–specific network contained 44 nodes (Figure A3A), with eight genes targeting more than 20 nodes. Meanwhile, the RWFC network had 48 nodes (Figure A3B), with no genes targeting more than 20 nodes.

These results reveal a substantial difference in the number of gene–gene interaction network nodes between the unique DEGs of WCCs and RWFCs. Specifically, the number of nodes induced by WCC–specific DEGs peaked on day 4 post–infection, while the RWFC–specific DEGs reached their peak at 2 dpi. Notably, several genes in WCCs, such as SLC7A11, CCL19, ACOX2, and HDAC1, may play a role in its relatively lower susceptibility to *E. tenella*. Therefore, future studies will focus on functional experiments on these hub genes both in vivo and in vitro to identify candidate genes associated with susceptibility to *E. tenella*.

### 3.8. Analysis of Overlapping DEGs in the Cecum of WCCs and RWFCs Following E. tenella Infection

**Figure 8:**
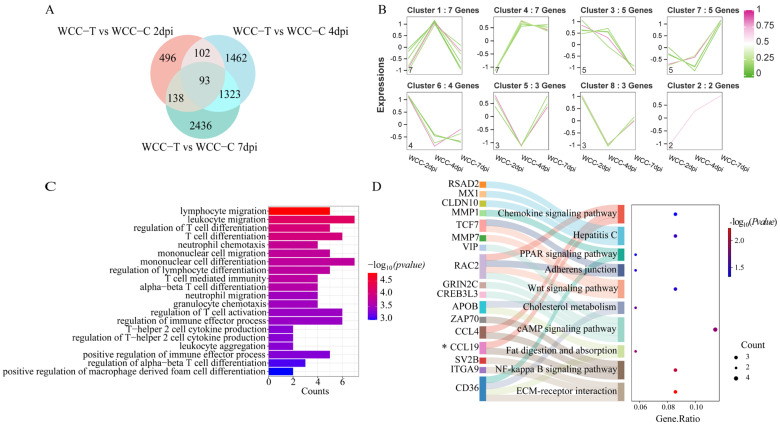
Analysis of overlapping DEGs following *E. tenella* infection in the WCC. (**A**) Venn diagram illustrating the overlap of DEGs in the WCC at 2 dpi (light orange, *n* = 829), 4 dpi (light blue, *n* = 2980), and 7 dpi (light green, *n* = 3990), resulting in 93 intersecting genes. (**B**) Fuzzy c-means clustering revealed eight distinct temporal expression patterns among the intersecting genes. The x-axis represents the three time points post-infection, while the y-axis shows log2-transformed and normalized intensity ratios. (**C**) The top 20 most significantly enriched GO pathways. (**D**) The Sankey plot illustrates the top 10 pathways with significantly enriched intersection genes in the WCC. A dot plot illustrates the proportion of WCC−T genes in relation to the total number of genes within each significantly enriched pathway (*p* ≤ 0.05). Genes marked with asterisks in the heatmap were selected for subsequent qRT−PCR validation.

**Figure 9:**
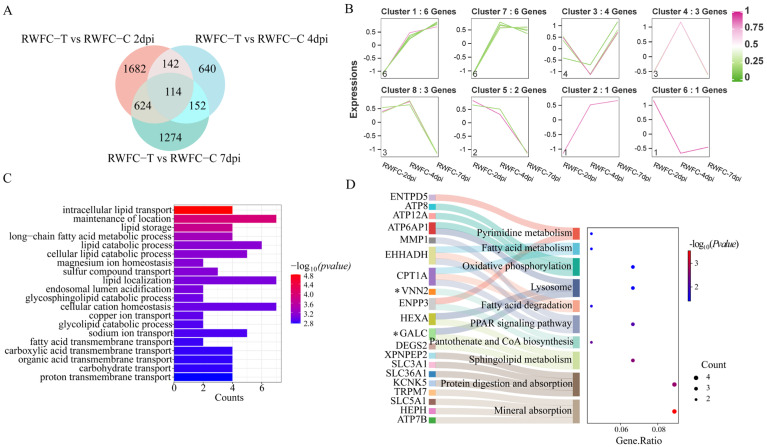
Analysis of overlapping genes following RWFC infection with *E. tenella*. (**A**) Venn diagram displays the overlapping of 2562 DEGs at 2 dpi (light orange), 1048 DEGs at 4 dpi (light blue), and 2164 DEGs at 7 dpi (light green), resulting in a total of 114 overlapping genes. (**B**) Trend analysis of the overlapping genes at 2, 4, and 7 days post-RWFC infection. The *x*-axis denotes the three time points following infection, while the *y*-axis illustrates the log2-transformed and normalized intensity ratios at each respective time point. (**C**) GO analysis of the top 20 most enriched GO pathways. (**D**) Sankey plot illustrating the top 10 significantly enriched pathways within the overlapping genes of the RWFC. The dot plot depicted the ratio of RWFC−T to RWFC−Cin relation to the total number of genes within each enriched pathway (*p* ≤ 0.05). Genes marked with asterisks in the heatmap were selected for subsequent qRT−PCR validation.

**Figure A4:**
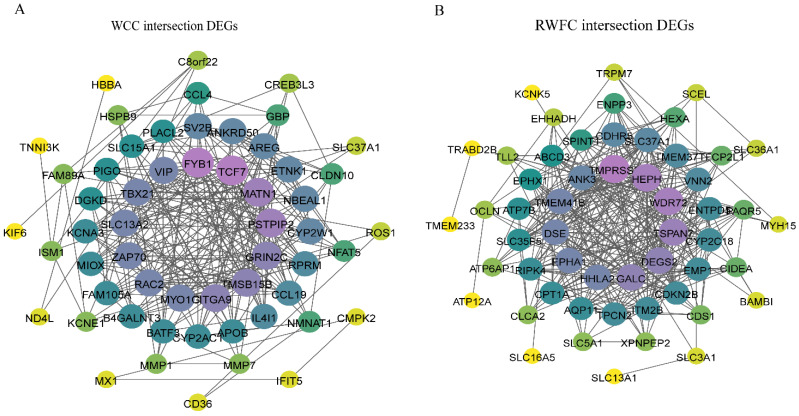
Gene–gene interaction network diagram illustrating the overlapping DEGs in WCCs and RWFCs after infection with *E. tenella*.

To further investigate the differences in host immune responses following *E. tenella* infection between WCCs and RWFCs, we analyzed the overlapping DEGs at three post-infection time points (2, 4, and 7 dpi) for each group (WCC−T vs. WCC−C and RWFC−T vs. RWFC−C). The results revealed that 93 overlapping DEGs were identified in the WCC group (Figure 8A), while 114 overlapping DEGs were detected in the RWFC group (Figure 9A). These overlapping DEGs were clustered into eight distinct expression patterns. In WCCs, cluster 3 comprised downregulated DEGs, while clusters 2 and 7 consisted of upregulated DEGs. Clusters 1, 4, 5, 6, and 8 included DEGs with a bi-modal expression pattern (Figure 8B). In RWFCs, clusters 5 and 8 contained downregulated DEGs, whereas clusters 1 and 2 contained upregulated DEGs. Clusters 3, 4, 6, and 7 exhibited a bi-modal expression pattern (Figure 9B). GO analysis of the overlapping DEGs in WCCs revealed significant associations with innate and adaptive immune responses, such as T cell differentiation, lymphocyte migration, and mononuclear cell differentiation (Figure 8C). KEGG analysis further highlighted significant enrichment in immune-related pathways, including ECM-receptor interaction, NF-kappa B signaling, and Wnt signaling (Figure 8D). In contrast, GO and KEGG analyses of the overlapping DEGs in RWFCs showed significant enrichment, primarily in lipid metabolism processes (Figure 9C,D). Gene–gene interaction network analysis of the WCC-overlapping DEGs revealed 56 nodes, with several genes, including MATN1, TCF7, PSTPIP2, and FYB1, interacting with more than 15 nodes (Figure A4A). The RWFC-overlapping DEGs consisted of 55 nodes, with 24 genes interacting with more than 15 nodes. Notably, genes such as TMPRSS2, HEPH, WDR72, TSPAN7, DEGS2, GALC, HHLA2, TMEM41B, DSE, and EPHA1 were identified as interacting with over 20 network nodes (Figure A4B).

In summary, the functional analysis of the overlapping genes suggests that the WCC–specific DEGs predominantly regulate immune responses, while those of RWFCs are more involved in lipid metabolism. Based on the findings from GO, KEGG, and gene–gene interaction network analyses, we infer that the stable and sustained immune response triggered by WCCs, combined with its distinct metabolic and functional alterations, likely contributes to its enhanced resistance to *E. tenella*.

### 3.9. Validation of Some Unique DEGs in the RNA−sequencing Results

**Figure 10:**
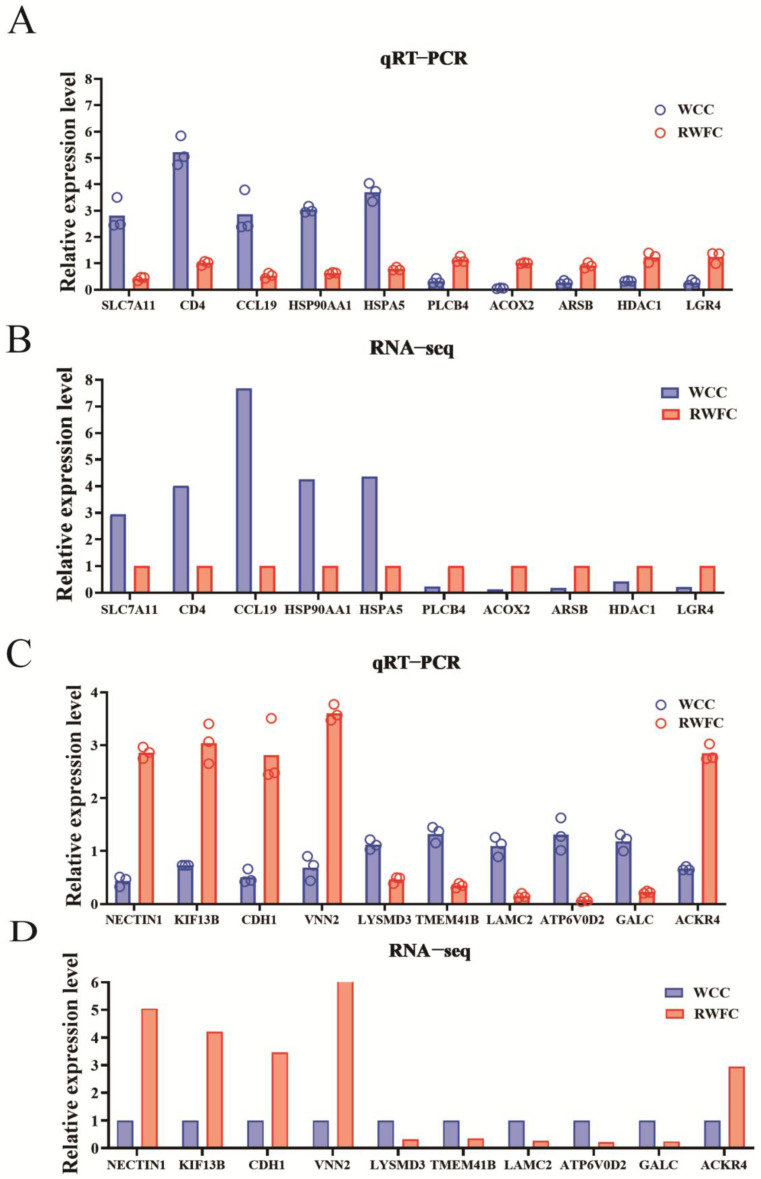
qRT−PCR validation was conducted on ten candidate-specific DEGs in each breed at three post-infection time points: 2, 4, and 7 dpi. (**A**) RNA extracted from WCCs and RWFCs, respectively, was used as a template to analyze the expression changes of specific DEGs selected from WCCs. (**B**,**D**) RNA−seq results of the selected specific DEGs were presented for comparison. (**C**) Changes in the specific DEGs selected from RWFCs were analyzed using RNA templates extracted from WCCs and RWFCs, respectively. The values indicate the mean fold change ± SE derived from three independent experiments.

To validate the DEGs specific to WCCs and RWFCs, we quantified the relative expression levels of 10 known genes in each breed using qRT−PCR. The target genes identified in WCCs included SLC7A11, CD4, CCL19, HSP90AA1, HSPA5, PLCB4, ACOX2, ARSB, HDAC1, and LGR4. In RWFCs, the selected target genes were NECTIN1, KIF13B, CDH1, VNN2, LYSMD3, TMEM41B, LAMC2, ATP6V0D2, GALC, and ACKR4. Except for VNN2 and GALC, these genes exhibited relatively high node degree values in the gene–gene interaction network. The results revealed that the expression profiles of the selected genes were consistent with the RNA sequencing data (Figure 10). These findings suggest that RNA−seq is a highly accurate and reliable method for investigating transcriptomic changes in WCCs and RWFCs following infection with *E. tenella*.

## 4. Discussion

Coccidiosis, caused by the genus *Eimeria*, is one of the major threats faced by the broiler chicken industry [25]. This study was designed to assess the relative susceptibility of WCCs and RWFCs to *E. tenella*-induced coccidiosis. The results indicated that WCCs exhibited a comparatively lower susceptibility to *E. tenella*, as evidenced by a reduced mortality rate and lower oocyst production compared to RWFCs (Figure 2A,D). While the average weight gain of RWFCs exceeded that of WCCs, the difference was not statistically significant (Figure 2B). This higher average weight gain in RWFCs may be attributed to their fast-growing nature, which resulted in a greater body weight prior to the *E. tenella* challenge [26]. As such, weight gain was not considered a primary indicator of coccidia susceptibility in this study. Based on these findings, this study demonstrated that, while WCCs exhibited marginally lower weight gain compared to RWFCs, they displayed a significantly greater resistance to disease. This conclusion aligns with the prevailing notion that local chicken breeds, which have not undergone extensive artificial selection, tend to possess enhanced disease resistance [27,28]. Therefore, to elucidate the mechanisms underlying the differential susceptibility to *E. tenella* between these two chicken breeds, this study systematically evaluated and compared the cellular immune responses, humoral immune responses, and cecal gene expression profiles following *E. tenella* infection. The primary objective was to identify key genes or signaling pathways contributing to the observed differences in susceptibility, thereby informing the development of effective strategies for the prevention and control of avian coccidiosis.

It is well-established that the immune system plays a pivotal role in determining the overall health of the host. Immune responses vary considerably across different host species, with T cell and B cell activities, antibody production, phagocytosis, and lymphocyte proliferation contributing to these differences [9,10]. Research has shown that *Eimeria* infection can trigger a robust immune response within the host, with both humoral and cellular immunity playing essential roles in establishing protective immunity against *Eimeria* [29]. The specific circulating antibodies produced during the humoral immune response, such as IgM, IgG, and IgA, are capable of inhibiting *Eimeria* invasion into host cells both in vivo and in vitro [30]. In our study, WCCs exhibited a significant elevation in IgG and IgA antibodies at 7 dpi (Figure 4A,B), suggesting that WCCs mount a more robust immune response to *E. tenella* infection compared to RWFCs, which likely explains their greater resistance to infection.

The cellular immune response is primarily mediated by thymus-dependent T lymphocytes, with CD4^+^ T helper cells (Ths) and CD8^+^ cytotoxic T lymphocytes (CTLs) being the two main subsets involved in anti-*Eimeria* immunity [31]. Regarding changes in T lymphocyte subpopulations, we observed that the levels of CD3^+^CD4^+^ T lymphocytes in WCCs were consistently higher than those in RWFCs, both pre- and post-infection (Figure 3B). In contrast, the levels of CD3^+^CD8α^+^ T lymphocytes in WCCs were lower than those in RWFCs (Figure 3C). This suggests that WCCs primarily eliminate pathogens by modulating immune responses and promoting cytotoxic activity, whereas RWFCs rely more on the direct destruction of infected target cells in cytotoxic responses. Notably, at 2 dpi, the levels of CD3^+^CD4^+^ T lymphocytes in WCCs were significantly higher compared to those in RWFCs (Figure 3B). This finding suggests that the relatively low susceptibility of WCCs facilitates a quicker initiation of CD4 Th cell-mediated immune responses to combat infection during the early stages. It can be inferred that CD4 Th cells may be key effectors contributing to the host’s resistance against *E. tenella* in the initial phase of infection. These findings align with those reported by Bremner et al. [4]. Furthermore, this study supports the conclusions of Dubey and Jenkins [32], which emphasize that host resistance to *E. tenella* is critically dependent on the host’s immune response in the first few days following infection.

To further explore the mechanisms behind the differences in susceptibility between WCCs and RWFCs following *E. tenella* infection, we employed RNA−seq technology to compare transcriptomic changes in cecal tissues of both breeds at 2, 4, and 7 dpi. Analysis revealed distinct gene expression profiles between the two breeds at various time points, consistent with the findings of Bremner et al. [4], who showed marked differences in gene expression profiles between the resistant C.B12 line and the susceptible 15I line during *E. maxima* infection. In this study, WCC DEGs exhibited an upregulated trend at 2 dpi and a declining trend from the 4th day onward (Figure 5), which may reflect an immunoregulatory mechanism that prevents excessive inflammatory responses.

KEGG pathway analysis revealed that WCCs exhibited a generally upward trend in the number of activated KEGG pathways and genes, while RWFCs showed fluctuations. Notably, compared to RWFCs, WCC–specific DEGs significantly activated several key immune-related pathways, including protein processing in the endoplasmic reticulum (ER), cytokine–cytokine receptor interaction, and ferroptosis (Figure 6). At 2 dpi, pathways such as focal adhesion, regulation of the actin cytoskeleton, and ECM–receptor interaction in RWFCs were significantly enriched. These pathways may work synergistically to facilitate pathogen-driven intracellular infection [33].

The ER is a crucial organelle involved in vesicular transport, calcium homeostasis, protein synthesis and folding, as well as lipid biosynthesis and metabolism [34]. Our study found that early infection with *E. tenella* induces ER stress, leading to functional alterations. This observation is consistent with the findings reported by Kim et al. [12]. Heat shock proteins (HSPs), such as HSP90AA1, HSP90B1, and HSPA5, which are involved in this pathway, were highly correlated and upregulated in the gene–gene interaction network (Figure 7A). Our findings are consistent with Sadr et al. [35], who demonstrated that local chicken breeds exhibit elevated levels of HSPs during infection. HSP90AA1 is a stress-inducible member of the heat shock protein 90 (HSP90) family and has been identified as a secreted extracellular factor involved in inflammatory responses [36]. Under pathophysiological conditions, HSPA5 can translocate from the ER to the nucleus, influencing gene transcription. Therefore, regulating the expression or functional activity of HSPA5 may offer a promising strategy for intervening in macrophage polarization across various disease states [37,38]. HSPA8, part of the HSPA/HSP70 family, is involved in autophagic protein degradation and antibacterial autophagy [39]. Therefore, we propose that the upregulation of HSPs in WCCs may be part of a cellular self-protective response to *E. tenella* infection, although the underlying mechanisms require further exploration.

Ferroptosis, which is closely linked to inflammatory responses, has emerged as a potential target for modulating inflammation [40]. SLC7A11, a key regulatory protein in this pathway, showed the strongest correlation in the gene–gene interaction network (Figure 6). Elevated expression levels of SLC7A11 are associated with resistance to ferroptosis, as this mechanism helps maintain intracellular glutathione levels, thereby protecting against oxidative damage [41]. Therefore, we speculate that, during the early stages of *E. tenella* infection, WCCs may regulate the inflammatory response through the ferroptosis pathway.

The cytokine–cytokine receptor interaction pathway in WCCs was significantly enriched at both 2 and 7 dpi, with genes such as CCL19, CCR7, CD4, and BMP4 exhibiting high correlation within the gene–gene interaction network (Figure 7A and Figure A3A). CCL19 is a key regulatory factor that mediates T cell activation, immune tolerance, and inflammatory responses during continuous immune surveillance, homeostasis, and development [42]. Additionally, the CCL19-CCR7 axis plays a crucial role in directing T cells and dendritic cells to lymphoid tissues, promoting T cell activation and initiating adaptive immune responses [43]. The CD4 molecule is essential for the development of CD4^+^ T lymphocytes, which serve as a pivotal component of the adaptive immune response. Upon pathogen infection, these cells proliferate and differentiate into effector and memory subsets, actively contributing to pathogen elimination [44]. BMP4, a member of the TGF-β superfamily, is involved in regulating morphogenesis, homeostasis, stem cell characteristics, and inflammatory responses in the gastrointestinal tract [45]. Based on these observations, it can be inferred that the inflammatory response in WCCs may be modulated through the cytokine–cytokine receptor interaction pathway following infection, thereby enhancing its capacity to resist infection. This finding is consistent with the conclusions of Vu et al. [46], which suggest that genes associated with cytokine–cytokine receptor interactions are more abundantly expressed in H5N1-resistant chickens. This increased gene expression enables resistant chickens to exhibit a stronger immune response and antiviral activity.

Although some hub genes were validated at the molecular level using qRT−PCR, functional experiments to confirm their biological roles have not been conducted. In future studies, we plan to systematically perform in vivo and in vitro functional validations focused on these key genes to further explore their biological significance. For example, the functional validation of the SLC7A11 gene will involve assessing glutathione content and intracellular cystine/cysteine levels.

Additionally, the enrichment of specific metabolic pathways in WCCs during *E. tenella* infection may contribute to their enhanced anti-infection capabilities. For example, lipid metabolism pathways, such as inositol phosphate metabolism, fatty acid metabolism, and glycerolipid metabolism, are notably enriched (Figure 6). Inositol and its phosphates serve as essential intracellular signaling molecules, playing pivotal roles in maintaining cellular homeostasis, antioxidant defense, and neurotransmission [47]. It has been reported that short-chain fatty acids (SCFAs) in fatty acid metabolism possess potent anti-inflammatory properties [48]. Among long-chain fatty acids (LCFAs), saturated fatty acids, trans fatty acids, and ω-6 polyunsaturated fatty acids have pro-inflammatory effects, while oleic acid and ω-3 polyunsaturated fatty acids exhibit anti-inflammatory properties [49]. Glyceride metabolism, which involves the synthesis and degradation of triglycerides and phospholipids, is crucial for maintaining cellular energy homeostasis and membrane integrity [50]. In light of these findings, it is highly plausible that WCCs enhance their anti-infection capabilities by modulating various lipid metabolic pathways during *E. tenella* infection.

Notably, the findings of this study reveal that the overlapping DEGs in WCCs are primarily involved in regulating immune responses, with significantly enriched pathways including ECM–receptor interaction, NF-κB signaling, cAMP signaling, and Wnt signaling (Figure 8C,D). In contrast, the DEGs in RWFCs are predominantly associated with lipid metabolism-related pathways, such as sphingolipid metabolism, PPAR signaling, and fatty acid metabolism (Figure 9C,D). The extracellular matrix (ECM) acts as a dynamic partner in immune regulation, consisting of secreted proteins such as cytokines, chemokines, and growth factors. These components are crucial in regulating immune cells and play essential roles in development, tissue formation, wound healing, and immune responses [51]. The NF-κB signaling pathway is central to both innate and adaptive immune responses, influencing the production of immunoregulatory proteins, anti-inflammatory cytokines, antimicrobial peptides, and other tolerance factors within the gut [52]. For many years, cAMP has been considered a key inducer of anti-inflammatory responses [53]. Additionally, studies have shown that mechanisms raising cellular cAMP levels can effectively suppress NF-κB activation through both the cAMP/PKA and cAMP/EPAC signaling cascades [54,55]. The canonical Wnt signaling pathway also demonstrates anti-inflammatory properties in various microbial infections, modulating the inflammatory responses of different cell types based on environmental factors, stimuli, or interactions with other signaling pathways [56]. Overall, upon infection with *E. tenella*, WCCs rapidly initiate both innate and adaptive immune responses to combat the infection. Subsequently, opposing mechanisms that inhibit lymphocyte activity are activated to exert anti-inflammatory effects. While lipid metabolism is involved in both WCCs and RWFCs, we hypothesize that the underlying regulatory mechanisms may differ. Specifically, lipid metabolism in WCCs promotes resistance to infection by modulating inflammatory responses, whereas in RWFCs, it appears to favor a pro-inflammatory direction.

## 5. Conclusions

In this study, we systematically compared the MR, BWG, OPG, and CLS in WCCs and RWFCs following infection with *E. tenella*. Our results revealed that, compared to RWFCs, WCCs exhibited relatively lower susceptibility to *E. tenella* infection. Additionally, the CD3^+^CD4^+^ T lymphocyte subset was consistently higher in WCCs, both prior to and after infection, whereas RWFCs displayed a higher quantity of CD3^+^CD8α^+^ T lymphocytes. Furthermore, WCCs elicited a more robust humoral immune response at 7 dpi compared to the control group. RNA−seq analysis further revealed that gene expression in WCCs showed a consistent upward trend following infection, whereas RWFCs exhibited a more fluctuating pattern. WCCs also consistently regulated genes associated with immune responses, while RWFCs tended to stimulate genes related to lipid metabolism. Moreover, several hub genes, including SLC7A11, CCL19, CD4, HSPA5, and HSP90AA1, along with pathways, such as protein processing in the endoplasmic reticulum, cytokine–cytokine receptor interaction, and ferroptosis, were highly associated with the differences observed between WCCs and RWFCs following *E. tenella* infection. Altogether, these findings provide valuable insights into the mechanisms underlying host susceptibility to *E. tenella*, which could be beneficial for the development of strategies for controlling and preventing coccidiosis.

## Data Availability

The raw data supporting the conclusions of this article will be made available by the authors upon request.

## Abbreviations

WCCs	Wenchang chickens
RWFCs	Recessive white feather chickens
DEGs	Differentially expressed genes
PBS	Phosphate-buffered saline
MR	Mortality rate
BWG	Body weight gain
OPG	Oocyst output per gram of feces
CLS	Cecal lesion score
dpi	Days post-infection
RIN	RNA integrity number
GO	Gene ontology
KEGG	Kyoto Encyclopedia of Genes and Genomes
FDR	False discovery rate
qRT−PCR	Quantitative reverse transcription polymerase chain reaction
MF	Molecular function
CC	Cellular component
BP	Biological process
Th	T helper cell
CTL	Cytotoxic T lymphocyte
ER	Endoplasmic reticulum
HSPs	Heat shock proteins
SCFAs	Short-chain fatty acids
LCFAs	Long-chain fatty acids

## Appendix A

**Table A1:** Laboratory sample analysis report.

No.	Sample	Sample No.	Residual Volume (μL)	Nanomolar Concentration (ng/μL)	Total Mass (μg)	OD260/280	OD260/230	RIN Value	Quality Inspection Conclusion
1	A4244	JB22ZNO00396	155.0	308.6	47.83	1.89	2.37	8.9	A
2	A4229	JB22ZNO00397	155.0	541.8	83.98	1.93	2.36	7.3	A
3	A3456	JB22ZNO00398	155.0	493.2	76.45	1.94	2.34	7.4	A
4	A3554	JB22ZNO00400	155.0	544.6	84.41	1.92	2.34	10.0	A
5	A2768	JB22ZNO00401	155.0	442.2	68.54	1.92	2.35	10.0	A
6	A3465	JB22ZNO00402	155.0	428.7	66.45	1.92	2.34	10.0	A
7	C021934	JB22ZNO00404	155.0	357.4	55.4	1.91	2.35	8.1	A
8	C021932	JB22ZNO00405	155.0	396.5	61.46	1.92	2.31	10.0	A
9	C021450	JB22ZNO00406	155.0	392.5	60.84	1.91	2.34	7.2	A
10	C021867	JB22ZNO00408	155.0	507.1	78.6	1.91	2.28	9.6	A
11	C022079	JB22ZNO00409	155.0	422.0	65.41	1.92	2.32	7.6	A
12	C021880	JB22ZNO00410	155.0	997.2	154.57	1.98	2.33	10.0	A
13	A4517	JB22ZNO00421	155.0	658.4	102.05	1.96	2.33	7.8	A
14	A4143	JB22ZNO00422	155.0	584.8	90.64	1.95	2.36	9.9	A
15	A4466	JB22ZNO00454	300.0	473.2	141.96	1.96	2.40	7.2	A
16	A2769	JB22ZNO00425	200.0	564.5	112.9	1.91	2.37	8.1	A
17	A4263	JB22ZNO00426	155.0	704.1	109.14	1.97	2.40	7.3	A
18	A4392	JB22ZNO00427	155.0	949.0	147.1	1.97	2.33	8.9	A
19	C021451	JB22ZNO00429	60.0	459.11	27.55	2.07	1.98	9.9	A
20	C021763	JB22ZNO00430	60.0	345.01	20.7	2.05	2.16	10.0	A
21	C021137	JB22ZNO00431	60.0	195.18	11.71	2.02	2.20	7.7	A
22	C021583	JB22ZNO00437	300.0	593.7	178.11	1.98	2.39	7.9	A
23	C021411	JB22ZNO00435	60.0	635.4	38.12	2.06	2.38	9.0	A
24	C022061	JB22ZNO00436	60.0	361.84	21.71	2.06	2.30	8.5	A
25	A4533	JB22ZNO00447	300.0	393.0	117.9	1.93	2.23	10.0	A
26	A4004	JB22ZNO00448	300.0	377.8	113.34	1.90	2.37	10.0	A
27	A3532	JB22ZNO00449	300.0	559.6	167.88	1.96	2.21	8.7	A
28	A4464	JB22ZNO00423	155.0	652.7	101.17	1.95	2.35	9.6	A
29	A4307	JB22ZNO00452	300.0	379.9	113.97	1.91	2.37	7.4	A
30	A3573	JB22ZNO00455	300.0	491.9	147.57	1.97	2.38	7.5	A
31	C021881	JB22ZNO00456	300.0	289.5	86.85	1.87	2.21	9.8	A
32	C022320	JB22ZNO00457	300.0	403.1	120.93	1.91	2.35	8.5	A
33	C021885	JB22ZNO00458	300.0	518.3	155.49	1.94	2.40	9.9	A
34	C021978	JB22ZNO00460	250.0	455.9	113.98	1.91	2.25	9.2	A
35	C021876	JB22ZNO00461	300.0	397.4	119.22	1.91	2.33	9.0	A
36	C021908	JB22ZNO00462	300.0	593.3	177.99	1.96	2.30	8.2	A

Quality grade A: The sample meets the required standards, and both its quality and total quantity are sufficient for subsequent library construction.

**Table A2:** Primer sequence used for qRT−PCR to validate DEGs identified in RNA−seq.

Primer Name	Primer Sequence (5′-3′)	Product Length (bp)	GenBank Accession No.
SLC7A11	TCGTGACGGTGCCTAATG	175	MH760782.1
	TTGGGTGAGATGAAGATGC		
CD4	TCCCTTTGTCCTGTTTCG	99	Y12012.1
	TCTTGTCCATTGGCTCCT		
CCL19	TATGCTGGCAACAACGTCCT	135	NM001302168.1
	TGCAGTGATGAACACGGTGG		
HSP90AA1	CGCAGCGTTGAGGGTGTA	85	NM001109785.2
	CACAGCTTCTGGCATCTTGG		
HSPA5	CCATTAAGGTCTATGAAGGTGAACG	224	NM205491.2
	CTCTGGTGTTAGCCGATTCT		
PLCB4	ATTCAAGCCATTAAGGAAAC	193	NM_001398213.1
	CATTAGGAGAAGGCAAGG		
ACOX2	CCTCCTGGGGATCATTCGG	165	XM046926551.1
	TAGGCTGGGCTTATCTGCTTGTT		
ARSB	TGGAGGACATACCAATGGCAC	146	XM_046936477.1
	TGTCAATGCCAAGACATGGGT		
HDAC1	GTCTGCTACTACTACGACGGTG	212	NM_204156.2
	AACAGCCCATCGAACACAGG		
LGR4	AGTCAGCCCACTCCGACTAT	101	XM_046919090.1
	TCGGCTTTGGTAGAAGCAGG		
NECTIN1	ATTTCAAGGGTCCTGTCTCC	249	XM_040690207.2
	GGTCATGGTCTGCCTCTGT		
KIF13B	GGCTACGGATTGCTTGTA	190	XM_040668432.2
	CTGTGGGTCCTCCTTCTG		
CDH1	CTCGGCAGAGGCAGCG	268	NM_001039258.3
	CTGCAAAGCTCACTCGTCCC		
VNN2	GGCATTTATGGCTGGGTT	126	NM_001379258.2
	TTCCTGCACTGGTGTTCG		
LYSMD3	TTTCAGTATAGACAGGAGGGTA	214	NM_001031414.2
	TAATCACAGCGAAGTGCC		
TMEM41B	TGGTCAGAACAGGTTGAGCG	221	NM_001008469.2
	TCCCCTGCTGTTGTAAGTTGG		
LAMC2	TGCTGCGTCTGATGAACC	158	XM_040677531.2
	TTTCTGTACCCGAACCTTCT		
ATP6V0D2	CAGCGACCCATAGCTGAACT	129	NM001008455.2
	AGCTAATGGCGTGTCAACCA		
GALC	CGATGTCATTGGGGCTCACT	140	XM_040672942.2
	GGTTTAGGATCCGAGCCCAG		
ACKR4	TGGGAACCTTTGTGCCAAGA	123	XM_040662558.2
	TGTGTCTCTGAAGCGGGAAC		

**Table A3:** Clean sequencing data statistics for thirty−six RNA−seq libraries.

No.	Sample	Total Reads	Total Beas	Clean Reads	Clean Ratio	No rRNA	rRNA Ratio	Q20	Q30
1	A2768	41290886	6193632900	40788174	0.98782511	40639184	0.003652774	97.15979161	93.71256892
2	A2769	43203532	6480529800	42829888	0.99135154	40363728	0.057580351	97.57117474	94.43186642
3	A3456	47722774	7158416100	47036118	0.985611566	45621216	0.030081181	96.96813771	93.13143801
4	A3465	42878956	6431843400	42423802	0.989385143	42311348	0.002650729	97.75753001	93.97439017
5	A3532	48303236	7245485400	47802900	0.989641771	47477980	0.006797077	97.3906402	94.33633048
6	A3554	47623442	7143516300	47073466	0.98845157	46927954	0.003091168	97.69168792	93.80712829
7	A3573	56306766	8446014900	55602814	0.987497915	55405198	0.003554065	97.07041633	93.59152257
8	A4004	44902512	6735376800	44461722	0.9901834	44281266	0.004058682	97.43871664	94.39259682
9	A4143	42843266	6426489900	42440050	0.990588579	42260616	0.00422794	97.50059303	94.50548856
10	A4229	47528730	7129309500	46951590	0.987857029	46520526	0.009181031	97.19146595	93.68076321
11	A4244	38980140	5847021000	38323006	0.983141826	37490376	0.021726636	97.15792741	93.44682431
12	A4263	39640364	5946054600	39172264	0.988191329	38236900	0.023878222	97.41623636	94.11710039
13	A4307	44204974	6630746100	43689548	0.98834009	43078204	0.013992912	97.23425705	93.84134892
14	A4392	42519718	6377957700	42093520	0.989976462	41502136	0.014049288	97.48222704	94.42762943
15	A4464	40600416	6090062400	40112294	0.987977414	39673088	0.010949411	97.3925364	94.10944045
16	A4466	46694404	7004160600	46013862	0.98542562	45845946	0.003649248	97.00504549	93.39408161
17	A4517	46574380	6986157000	46047940	0.98869679	45648192	0.008681127	97.35008101	94.01003127
18	A4533	42679370	6401905500	42265082	0.990293015	42086276	0.004230584	97.43959975	94.37898149
19	C021137	49039190	7355878500	48484036	0.988679381	47655404	0.017090821	97.23522776	93.7609628
20	C021411	43741574	6561236100	43264444	0.989092071	40508214	0.063706585	97.25531608	93.70296684
21	C021450	47319258	7097888700	46746086	0.98788713	46167824	0.012370276	97.11530885	93.45446355
22	C021451	43562000	6534300000	43142000	0.990358569	42339150	0.018609476	97.47991956	94.42757067
23	C021583	47628328	7144249200	47094544	0.98879272	46711880	0.008125442	97.24236733	93.78820254
24	C021763	44549006	6682350900	44073958	0.989336507	43712598	0.008198946	97.81448294	94.0684538
25	C021867	52064670	7809700500	51472932	0.988634558	50792016	0.013228623	97.24649714	93.94987346
26	C021876	47505956	7125893400	46826836	0.985704529	46494220	0.007103106	96.87017573	92.93656506
27	C021880	46751736	7012760400	46262020	0.98952518	46124444	0.002973843	97.79330031	94.1210958
28	C021881	46247662	6937149300	45677094	0.987662771	45528750	0.003247667	97.65677164	93.89365084
29	C021885	45717196	6857579400	45198398	0.988652016	45034068	0.003635748	97.68713207	93.93626766
30	C021908	46521638	6978245700	45906050	0.986767706	45574782	0.007216217	96.97610112	93.34343283
31	C021932	42264136	6339620400	41762602	0.988133343	41624064	0.003317274	97.19962902	93.77237577
32	C021934	46716960	7007544000	46144728	0.987751087	45945478	0.004317936	97.1564588	93.64715317
33	C021978	46890136	7033520400	46252006	0.986390954	45999846	0.005451872	97.03469799	93.49602634
34	C022061	37672330	5650849500	37146066	0.98603049	36569664	0.015517175	97.13091751	93.40916687
35	C022079	49964930	7494739500	49291222	0.986516383	48962380	0.006671411	97.04056102	93.35227813
36	C022320	42161842	6324276300	41699750	0.989040042	41553490	0.003507455	97.71758185	94.00582674

**Table A4:** Mapped data obtained from thirty−six RNA−seq libraries.

No.	Sample	Total_Reads	Mapped_Reads	Pair_Mapped_Reads	Single_Mapped_Reads	Unique_Mapped_Reads	Multi_Mapped_Reads	Mapped_Ratio
1	A2768	40639184	37648050	36856456	791594	37648050	0	0.926397784
2	A2769	40363728	37136457	36607972	528485	37136457	0	0.920045269
3	A3456	45621216	42549740	41693572	856168	42549740	0	0.932674394
4	A3465	42311348	39546101	38729866	816235	39546101	0	0.934645263
5	A3532	47477980	44601659	43667408	934251	44601659	0	0.939417789
6	A3554	46927954	43783548	42959934	823614	43783548	0	0.932995033
7	A3573	55405198	51353274	50245204	1108070	51353274	0	0.926867439
8	A4004	44281266	41224141	40452846	771295	41224141	0	0.930961211
9	A4143	42260616	39444407	38666896	777511	39444407	0	0.93336091
10	A4229	46520526	43670452	42921832	748620	43670452	0	0.93873513
11	A4244	37490376	35070310	34471096	599214	35070310	0	0.93544834
12	A4263	38236900	33282278	32793024	489254	33282278	0	0.870423021
13	A4307	43078204	39293524	38376134	917390	39293524	0	0.91214397
14	A4392	41502136	38637784	37964146	673638	38637784	0	0.930983022
15	A4464	39673088	37165180	36472096	693084	37165180	0	0.936785662
16	A4466	45845946	41188366	40215890	972476	41188366	0	0.898408029
17	A4517	45648192	42749198	41948938	800260	42749198	0	0.936492687
18	A4533	42086276	39455760	38808252	647508	39455760	0	0.93749706
19	C021137	47655404	44757529	43960204	797325	44757529	0	0.939191052
20	C021411	40508214	35861255	35372872	488383	35861255	0	0.885283538
21	C021450	46167824	43457350	42595930	861420	43457350	0	0.941290844
22	C021451	42339150	39501327	38742672	758655	39501327	0	0.93297402
23	C021583	46711880	37963792	37300058	663734	37963792	0	0.812722417
24	C021763	43712598	40986368	40234750	751618	40986368	0	0.937632854
25	C021867	50792016	47449270	46505334	943936	47449270	0	0.93418757
26	C021876	46494220	41612642	40456134	1156508	41612642	0	0.895006777
27	C021880	46124444	43005919	42131352	874567	43005919	0	0.932388887
28	C021881	45528750	42528020	41639618	888402	42528020	0	0.934091536
29	C021885	45034068	41875304	40997744	877560	41875304	0	0.929858346
30	C021908	45574782	41179602	40222118	957484	41179602	0	0.90356114
31	C021932	41624064	38877768	38106328	771440	38877768	0	0.934021435
32	C021934	45945478	42834059	42037960	796099	42834059	0	0.932280191
33	C021978	45999846	41358882	40363442	995440	41358882	0	0.89910914
34	C022061	36569664	32455229	31969348	485881	32455229	0	0.887490489
35	C022079	48962380	45904961	44905224	999737	45904961	0	0.937555752
36	C022320	41553490	39033181	38230696	802485	39033181	0	0.939347838
